# On the Extended Generalized Inverted Kumaraswamy Distribution

**DOI:** 10.1155/2022/1612959

**Published:** 2022-02-17

**Authors:** Qasim Ramzan, Sadia Qamar, Muhammad Amin, Huda M. Alshanbari, Amna Nazeer, Ahmed Elhassanein

**Affiliations:** ^1^Department of Statistics, University of Sargodha, Sargodha, Pakistan; ^2^Department of Mathematical Sciences, College of Science, Princess Nourah bint Abdulrahman University, P.O. Box 84428, Riyadh 11671, Saudi Arabia; ^3^Department of Mathematics, COMSATS University Islamabad, Islamabad, Pakistan; ^4^Department of Mathematics, College of Science, University of Bisha, Bisha, Saudi Arabia; ^5^Department of Mathematics, Faculty of Science, Damanhour University, Damanhour, Egypt

## Abstract

In this work, we provide a new generated class of models, namely, the extended generalized inverted Kumaraswamy generated (EGIKw-G) family of distributions. Several structural properties (survival function (sf), hazard rate function (hrf), reverse hazard rate function (rhrf), quantile function (qf) and median, *s*^th^ raw moment, generating function, mean deviation (md), etc.) are provided. The estimates for parameters of new *G* class are derived via maximum likelihood estimation (MLE) method. The special models of the proposed class are discussed, and particular attention is given to one special model, the extended generalized inverted Kumaraswamy Burr XII (EGIKw-Burr XII) model. Estimators are evaluated via a Monte Carlo simulation (MCS). The superiority of EGIKw-Burr XII model is proved using a lifetime data applications.

## 1. Introduction

Study of data is the most important and fundamental topic in statistics. The probability distributions help in the characterization of the variability and uncertainty prevailing in data by identifying the patterns of variation. The objective of statistical modeling is to develop appropriate probability distributions that adequately explain a data set generated by surveys, observational studies, experiment, etc.

In this context, there have been fundamental and significant thriving in probability distribution theory via the introduction of new generalized families of distributions, and several techniques to develop new distributions have been proposed. Some well-known systems of distributions are the beta generalized family of distributions by Eugene et al. [1], gamma generalized family by Zografos and Balakrishnan [2], Kumaraswamy generalized class of distributions by Cordeiro and de Castro [3], McDonald generalized family by Al-Sarabia [2012], gamma generalized family of distributions (type 2) by Ristic and Balakishnan [4], gamma generalized family (type 3) by Torabi and Hedesh [5], transformed-transformer (T-X) family by Alzaatreh et al. [6], logistic generalized family of distributions by Torabi and Montazeri [7], Weibull generalized class by Bourguignon et al. [8], Lomax generalized family of distributions by Cordeiro et al. [9], logistic *X* by Tahir et al. [10], odd generalized exponential family (OGE-G) by Tahir et al. [11], Garhy generalized class by Elgarhy et al. [12], Kumaraswamy–Weibull generalized family of distributions by Hassan and Elgarhy [13], exponentiated Weibull generalized family by Hassan and Elgarhy [14], additive Weibull generalized family by Hassan and Hemeda [15], type II half logistic generalized class by Hassan et al. [16], Zubair-G family of distributions by Ahmad [17], generalized inverted Kumaraswamy (GIKw) generated class by Jamal et al. [18], exponentiated Kumaraswamy-G class by Silva et al. [19], and type II Kumaraswamy half logistic family by El-Sherpieny and Elsehetry [20].

The inverted distributions are applied in various spheres of life including life testing, biology, environmental science, engineering sciences, and econometrics. Al-Fattah et al. [21] proposed the inverted Kumaraswamy (IKw) model via *Y* = 1/*X* − 1 transformation, when *X* has a Kumaraswamy distribution. Iqbal et al. [22] further generalized the model via transformation *T* =  *X*^*γ*^ to introduce the IKw distribution and proposed the generalized inverted Kumaraswamy (GIKum) distribution with respective cdf and pdf:

(1) $$\begin{array}{c} F\left(x\right)={\left[1-{\left(1+x^{\gamma }\right)}^{-\alpha }\right]}^{\beta },\\ f\left(x\right)=\alpha \beta \gamma x^{\gamma -1}{\left(1+x\right)}^{-\alpha -1}{\left[1-{\left(1+x^{\gamma }\right)}^{-\alpha }\right]}^{\beta -1}, \end{array}$$

where *α* > 0,  *β* > 0,  *γ* > 0 are the shape parameters, and *x* > 0. Let *s*(*t*) denote the expression for pdf of some random variable (rv), *T* ∈ [*a*, *b*], where −*∞* ≤ *a* < *b* < *∞*, and consider *D*[*W*(*x*)] is some function of cdf of another *rv*, say *X*; the *T*-*X* family can be defined as

(2) $$\begin{array}{c} F\left(x\right)=\int_{a}^{D\left[W\left(x\right)\right]}s\left(t\right)\text{d}t, \end{array}$$

where *D*[*W*(*x*)] satisfies the following:

1. *D*[*W*(*x*)] ∈ [*a*, *b*].
2. *D*[*W*(*x*)] is differentiable and monotonically nondecreasing function.
3. *D*[*W*(*x*)]⟶*a* as *x*⟶−*∞*, *D*[*W*(*x*)]⟶*b* as *x*⟶*∞*.

We give a new G class, the extended generalized inverted Kumaraswamy generated (EGIKw-G) family, considering *s*(*t*) to be GIKum and using the generator (*W*^*λ*^(*x*, *ϑ*)/1 − *W*^*λ*^(*x*, *ϑ*)) as *D*[*W*(*x*)] in (2) in order to obtain the distributions which show higher flexibility compared with other commonly used standard distributions; see [23, 24]. For *W*(*x*) some baseline cdf, the expression for the cdf of EGIKw-G class is

(3) $$\begin{array}{c} F_{\mathrm{EGIKw-G}}\left(x\right)=\alpha \beta \gamma \int_{0}^{\left(W^{\lambda }\left(x,\vartheta \right)/1-W^{\lambda }\left(x,\vartheta \right)\right)}t^{\gamma -1}{\left(1+t\right)}^{-\alpha -1}{\left[1-{\left(1+t^{\gamma }\right)}^{-\alpha }\right]}^{\beta -1}dt\text{,} \end{array}$$

or equivalently

(4) $$\begin{array}{c} F_{\mathrm{EGIKw-G}}\left(x\right)={\left\{1-{\left[1+{\left(\frac{W^{\lambda }\left(x,\vartheta \right)}{1-W^{\lambda }\left(x,\vartheta \right)}\right)}^{\gamma }\right]}^{-\alpha }\right\}}^{\beta }, \end{array}$$

where *α* > 0,  *β* > 0,  *λ*  and *γ* > 0 are extra positive parameters which offer the skewness, hence promoting the tails weight variation, and *ϑ* denotes baseline parametric space. For the conditions on baseline distributions, a detailed note can be found in Alzaatreh et al. [6]. In the following section, the pdf, reliability measures, and qf are explored. In Section 3, four special submodels of EGIKw-G class are discussed. In Section 4, several useful properties of the suggested class are provided. In Section 5, MCS study and MLEs are considered to verify the convergence properties. In Section 6, the practical importance of considered G class is examined through real-word data.

## 2. Density and Reliability Measures

In this part of paper, we offer a brief discussion on some of the other basic functions related to the EGIKw-G class of models including the pdf, the sf, the hrf, the rhrf, and the cumulative hazard rate function (chrf) which have an important role in reliability theory. If *X* follows EGIKw-G class (4), then its pdf is

(5) $$\begin{array}{c} f_{\text{EGIKw}-\text{G}}\left(x\right)=\alpha \beta \gamma \lambda w\left(x,\vartheta \right)\frac{W^{\lambda -1}\left(x,\vartheta \right)}{{\left[1-W^{\lambda }\left(x,\vartheta \right)\right]}^{2}}{\left(\frac{W^{\lambda }\left(x,\vartheta \right)}{1-W^{\lambda }\left(x,\vartheta \right)}\right)}^{\gamma -1}\times {\left[1+{\left(\frac{W^{\lambda }\left(x,\vartheta \right)}{1-W^{\lambda }\left(x,\vartheta \right)}\right)}^{\gamma }\right]}^{-\alpha -1}\times {\left\{1-{\left[1+{\left(\frac{W^{\lambda }\left(x,\vartheta \right)}{1-W^{\lambda }\left(x,\vartheta \right)}\right)}^{\gamma }\right]}^{-\alpha }\right\}}^{\beta -1}\\ =\alpha \beta \gamma \lambda w\left(x,\vartheta \right)W^{\gamma \lambda -1}\left(x,\vartheta \right){\left[1-W^{\lambda }\left(x,\vartheta \right)\right]}^{-\gamma -1}\times {\left[1+{\left(\frac{W^{\lambda }\left(x,\vartheta \right)}{1-W^{\lambda }\left(x,\vartheta \right)}\right)}^{\gamma }\right]}^{-\alpha -1}\times {\left\{1-{\left[1+{\left(\frac{W^{\lambda }\left(x,\vartheta \right)}{1-W^{\lambda }\left(x,\vartheta \right)}\right)}^{\gamma }\right]}^{-\alpha }\right\}}^{\beta -1}. \end{array}$$

The expressions for the sf, the hrf, the rhrf, and the chrf are given by

(6) $$\begin{array}{c} S_{\text{EGIKw}-\text{G}}\left(x\right)=1-{\left\{1-{\left[1+{\left(\frac{W^{\lambda }\left(x,\vartheta \right)}{1-W^{\lambda }\left(x,\vartheta \right)}\right)}^{\gamma }\right]}^{-\alpha }\right\}}^{\beta },\\ h_{\text{EGIKw}-\text{G}}\left(x\right)=\alpha \beta \gamma \lambda w\left(x,\vartheta \right)W^{\gamma \lambda -1}\left(x,\vartheta \right){\left[1-W^{\lambda }\left(x,\vartheta \right)\right]}^{-\gamma -1}\\ \times {\left[1+{\left(\frac{W^{\lambda }\left(x,\vartheta \right)}{1-W^{\lambda }\left(x,\vartheta \right)}\right)}^{\gamma }\right]}^{-\alpha -1}\\ \times {\left\{1-{\left[1+{\left(\frac{W^{\lambda }\left(x,\vartheta \right)}{1-W^{\lambda }\left(x,\vartheta \right)}\right)}^{\gamma }\right]}^{-\alpha }\right\}}^{\beta -1}\\ \times {\left[1-{\left\{1-{\left[1+{\left(\frac{W^{\lambda }\left(x,\vartheta \right)}{1-W^{\lambda }\left(x,\vartheta \right)}\right)}^{\gamma }\right]}^{-\alpha }\right\}}^{\beta }\right]}^{-1},\\ H_{\text{EGIKw}-\text{G}}\left(x\right)=\alpha \beta \gamma \lambda w\left(x,\vartheta \right)W^{\gamma \lambda -1}\left(x,\vartheta \right){\left[1-W^{\lambda }\left(x,\vartheta \right)\right]}^{-\gamma -1}\\ \times {\left[1+{\left(\frac{W^{\lambda }\left(x,\vartheta \right)}{1-W^{\lambda }\left(x,\vartheta \right)}\right)}^{\gamma }\right]}^{-\alpha -1}\times {\left\{1-{\left[1+{\left(\frac{W^{\lambda }\left(x,\vartheta \right)}{1-W^{\lambda }\left(x,\vartheta \right)}\right)}^{\gamma }\right]}^{-\alpha }\right\}}^{-1},\\ \Omega_{\text{EGIKw}-\text{G}}\left(x\right)=-\log\left[1-F\left(x\right)\right]\\ =-\log\left[1-{\left\{1-{\left[1+{\left(\frac{W^{\lambda }\left(x,\vartheta \right)}{1-W^{\lambda }\left(x,\vartheta \right)}\right)}^{\gamma }\right]}^{-\alpha }\right\}}^{\beta }\right], \end{array}$$

respectively. The EGIKw-G class can be easily simulated through inverting (4) as follows: let *u* be a standard uniform rv, rv; the inverse cdf or qf is given by solving *F*_EGIKw−G_(*x*_*u*_)=*u* as

(7) $$\begin{array}{c} Q\left(u\right)=x_{u}\\ =W^{-1}{\left[1+{\left[{\left(1-u^{\left(1/\beta \right)}\right)}^{-\left(1/\alpha \right)}-1\right]}^{-\left(1/\gamma \right)}\right]}^{-\left(1/\lambda \right)}. \end{array}$$

Furthermore, median, three quartiles, and seven octiles can be, respectively, obtained by *Q*(0.5), *Q*(0.5); *q*_*i*_=*Q*(*i*/4),  *i* ∈ (1,2,3); and *O*_*j*_=*Q*(*j*/8),  *j* ∈ (1,2,3,4,5,6,7). The qf is useful for evaluating some crucial properties including skewness, kurtosis, and central probabilistic results. The Bowley skewness is given by

(8) $$\begin{array}{c} S_{kb}=\frac{q_{3}+q_{1}-2q_{2}}{q_{3}-q_{1}}. \end{array}$$

For some baseline distribution *W*(*x*) when the resulting EGIKw-G distribution is symmetric, right skewed, and left skewed, we have *S*_*k*_ = 0, *S*_*k*_ > 0, and *S*_*k*_ < 0, respectively. A measure of kurtosis, the Moors kurtosis (see, e.g., Moors [25]), is given as

(9) $$\begin{array}{c} K_{um}=\frac{O_{3}-O_{1}+O_{7}-O_{5}}{O_{6}-O_{2}}. \end{array}$$

The tail of the EGIKw-G distribution becomes heavier as *K*_*um*_ increases, provided that *W*(*x*), *α*, *β*, *γ*,  and *λ* remain unchanged.

Note that the EGIKw-G class of models outlined above reduces to generalized inverted Kumaraswamy generated (EGIKw-G) class proposed by Jamal et al. [18], for *γ*=1, and when *γ*=1,  *λ*=1, the exponentiated-G class given by Cordeiro et al. [26] is obtained. Hence, parameter *γ* offers more flexibility to the extremes for the density function curves, and therefor new G class becomes more suitable for data sets which exhibit heavy tail. For every generated model, *“W”* and *“w”* represent baseline cdf and pdf, respectively.

## 3. Special Models

The EGIKw-G density function (4) offers high flexibility in tails along with promoting variation in tail weights to extremes of specific model. In this section, we provide four of many possible submodels under EGIKw-G class offering a more better fit to the data. For brevity, in the remainder of this paper, we shall comment in detail on only four of the most impotent EGIKw-G distributions, namely, EGIKw-Normal, EGIKw-Fréchet, EGIKw-Uniform, and EGIKw-Burr XII distributions.

### 3.1. EGIKw-Normal Distribution

**Figure 1:**
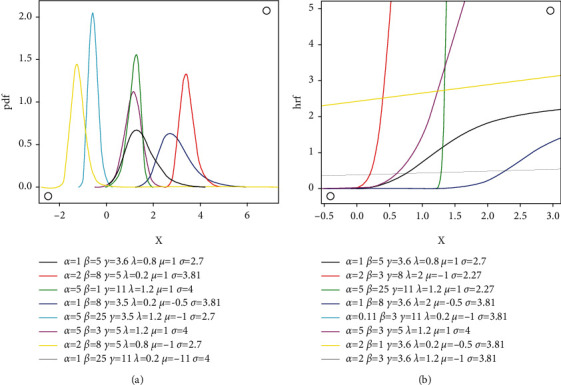
Selected *f*(*x*) and *h*(*x*) graphs of EGIKw-Normal model.

The EGIKw-Normal pdf is obtained from (5) for *W*(*x*)=Φ((*x* − *μ*)/*σ*) and *W*(*x*)=*ϕ*((*x* − *μ*)/*σ*), so

(10) $$\begin{array}{c} f\left(x\right)=\alpha \beta \gamma \lambda \frac{1}{\sigma }\phi \left(\frac{x-\mu }{\sigma }\right){\left[\Phi \left(\frac{x-\mu }{\sigma }\right)\right]}^{\lambda \gamma -1}\\ \times {\left[1-{\left[\Phi \left(\frac{x-\mu }{\sigma }\right)\right]}^{\lambda }\right]}^{-\gamma -1}\\ \times {\left\{1+{\left(\frac{{\left[\Phi \left(\left(x-\mu \right)/\sigma \right)\right]}^{\lambda }}{1-{\left[\Phi \left(\left(x-\mu \right)/\sigma \right)\right]}^{\lambda }}\right)}^{\gamma }\right\}}^{-\alpha -1}\\ \times {\left[1-{\left\{1+{\left(\frac{{\left[\Phi \left(\left(x-\mu \right)/\sigma \right)\right]}^{\lambda }}{1-{\left[\Phi \left(\left(x-\mu \right)/\sigma \right)\right]}^{\lambda }}\right)}^{\gamma }\right\}}^{-\alpha }\right]}^{\beta -1}\text{,} \end{array}$$

and the cdf is

(11) $$\begin{array}{c} F\left(x\right)={\left[1-{\left\{1+{\left(\frac{{\left[\Phi \left(\left(\left(x-\mu \right)/\sigma \right)\right)\right]}^{\lambda }}{1-{\left[\Phi \left(\left(\left(x-\mu \right)/\sigma \right)\right)\right]}^{\lambda }}\right)}^{\gamma }\right\}}^{-\alpha }\right]}^{\beta }, \end{array}$$

where *x* ∈ *ℝ*,  *μ* ∈ *ℝ*, and *σ* > 0; *ϕ*(·) and Φ(·), respectively, denote the standard normal pdf and cdf. The *rvX* in the above expression is EGIKw-N, e.g., *X* ~ EGIKw − *N*(*α*, *β*, *γ*, *λ*, *μ*, *σ*^2^).  For *μ*=0  and  *σ*=1, it reduces to standard EGIKw-N distribution. The pdf and hrf plots of EGIKw-N model are depicted in Figure 1. As given in Figure 1(b), the hrf gives increasing, inverted bathtub, or decreasing (reversed-J) shapes.

### 3.2. EGIKw-Fréchet Distribution

The Fréchet cdf and pdf for *x*>*rbin* 0, *δ* > 0 , and  *φ* > 0  are  *W*(*x*)=*exp*(−*δx*^−*φ*^)  and  *w*(*x*)=*δφx*^−*φ*−1^exp(−*δx*^−*φ*^), respectively. Correspondingly, the EGIKw-Fréchet EGIKw − F(*α*, *β*, *γ*, *λ*, *δ*, *ϕ*) is

(12) $$\begin{array}{c} f\left(x\right)=\alpha \beta \gamma \lambda \delta \varphi x^{-\varphi -1}\exp\left(-\delta x^{-\varphi }\right){\left[\exp\left(-\delta x^{-\varphi }\right)\right]}^{\lambda \gamma -1}\\ \times {\left[1-{\left[\exp\left(-\delta x^{-\varphi }\right)\right]}^{\lambda }\right]}^{-\gamma -1}\\ \times {\left\{1+{\left(\frac{{\left[\exp\left(-\delta x^{-\varphi }\right)\right]}^{\lambda }}{1-{\left[\exp\left(-\delta x^{-\varphi }\right)\right]}^{\lambda }}\right)}^{\gamma }\right\}}^{-\alpha -1}\\ \times {\left[1-{\left\{1+{\left(\frac{{\left[\exp\left(-\delta x^{-\varphi }\right)\right]}^{\lambda }}{1-{\left[\exp\left(-\delta x^{-\varphi }\right)\right]}^{\lambda }}\right)}^{\gamma }\right\}}^{-\alpha }\right]}^{\beta -1}. \end{array}$$

**Figure 2:**
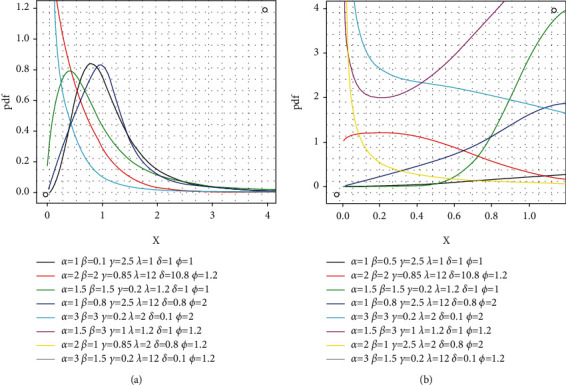
Selected *f*(*x*) and *h*(*x*) graphs for EGIKw-Fréchet model.

The cdf is

(13) $$\begin{array}{c} F\left(x\right)={\left[1-{\left\{1+{\left(\frac{{\left[\exp\left(-\delta x^{-\varphi }\right)\right]}^{\lambda }}{1-{\left[\exp\left(-\delta x^{-\varphi }\right)\right]}^{\lambda }}\right)}^{\gamma }\right\}}^{-\alpha }\right]}^{\beta }, \end{array}$$

where *x*, *α*, *β*, *γ*, *λ*, *δ*, *φ* > 0. For *φ*=1, we obtain the extended generalized inverted Kumaraswamy inverse exponential distribution.  Figure 2(a) indicates that the EGIKw-Fréchet offers various interesting shapes.  Figure 2(b) reveals that the model can also offer various hrf shapes including decreasing, increasing, *J*, revered-J, and bathtub shapes.

### 3.3. EGIKw-Uniform Distribution

The EGIKw-U pdf is obtained from (5), taking *W*(*x*)=(*x*/*θ*)  and  *w*(*x*)=(1/*θ*), where *x* ∈ (0, *θ*), as follows:

(14) $$\begin{array}{c} f\left(x\right)=\alpha \beta \gamma \lambda \frac{1}{\sigma }\phi \left(\frac{x-\mu }{\sigma }\right){\left[\frac{x}{\theta }\right]}^{\lambda \gamma -1}\\ \times {\left[1-{\left[\frac{x}{\theta }\right]}^{\lambda }\right]}^{-\gamma -1}{\left\{1+{\left(\frac{{\left[x/\theta \right]}^{\lambda }}{1-{\left[x/\theta \right]}^{\lambda }}\right)}^{\gamma }\right\}}^{-\alpha -1}\\ \times {\left[1-{\left\{1+{\left(\frac{{\left[x/\theta \right]}^{\lambda }}{1-{\left[x/\theta \right]}^{\lambda }}\right)}^{\gamma }\right\}}^{-\alpha }\right]}^{\beta -1}. \end{array}$$

The cdf is

(15) $$\begin{array}{c} F\left(x\right)={\left[1-{\left\{1+{\left(\frac{{\left[x/\theta \right]}^{\lambda }}{1-{\left[x/\theta \right]}^{\lambda }}\right)}^{\gamma }\right\}}^{-\alpha }\right]}^{\beta }\text{.} \end{array}$$

**Figure 3:**
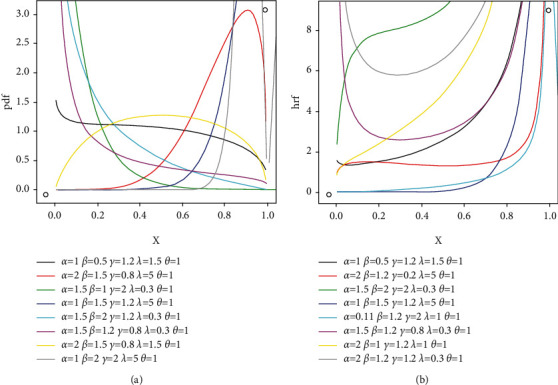
Selected *f*(*x*) and *h*(*x*) graphs for EGIKw-Uniform model.

A rv, say *X*, with above model is given as *X* ~ EGIKw − U(*α*, *β*, *γ*, *λ*, *θ*). For *θ*=1, we have standard EGIKw-Uniform model.  Figure 3 illustrates shapes of pdf and hrf for the EGIKw-Uniform model. The pdf plot in Figure 3(a) offers a variety of shapes. Moreover, it is obvious from Figure 3(b) that this model can accommodate constant, decreasing, and unimodal hrf.

### 3.4. EGIKw-Burr XII Distribution

The Burr XII pdf and cdf are *w*(*x*)=*ψξx*^*ξ*−1^(1+*x*^*ξ*^)^−*ψ*−1^ and *W*(*x*)=1 − (1+*x*^*ξ*^)^−*ψ*^, respectively. Hence, the EGIKw-Burr XII pdf is

(16) $$\begin{array}{c} f\left(x\right)=\alpha \beta \gamma \lambda \psi \xi x^{\xi -1}{\left(1+x^{\xi }\right)}^{-\psi -1}{\left[1-{\left(1+x^{\xi }\right)}^{-\psi }\right]}^{\lambda \gamma -1}\\ \times {\left[1-{\left[1-{\left(1+x^{\xi }\right)}^{-\psi }\right]}^{\lambda }\right]}^{-\gamma -1}\\ \times {\left\{1+{\left(\frac{{\left[1-{\left(1+x^{\xi }\right)}^{-\psi }\right]}^{\lambda }}{1-{\left[1-{\left(1+x^{\xi }\right)}^{-\psi }\right]}^{\lambda }}\right)}^{\gamma }\right\}}^{-\alpha -1}\\ \times {\left[1-{\left\{1+{\left(\frac{{\left[1-{\left(1+x^{\xi }\right)}^{-\psi }\right]}^{\lambda }}{1-{\left[1-{\left(1+x^{\xi }\right)}^{-\psi }\right]}^{\lambda }}\right)}^{\gamma }\right\}}^{-\alpha }\right]}^{\beta -1}. \end{array}$$

The corresponding cdf takes the following form:

(17) $$\begin{array}{c} F\left(x\right)={\left[1-{\left\{1+{\left(\frac{{\left[1-{\left(1+x^{\xi }\right)}^{-\psi }\right]}^{\lambda }}{1-{\left[1-{\left(1+x^{\xi }\right)}^{-\psi }\right]}^{\lambda }}\right)}^{\gamma }\right\}}^{-\alpha }\right]}^{\beta }. \end{array}$$

**Figure 4:**
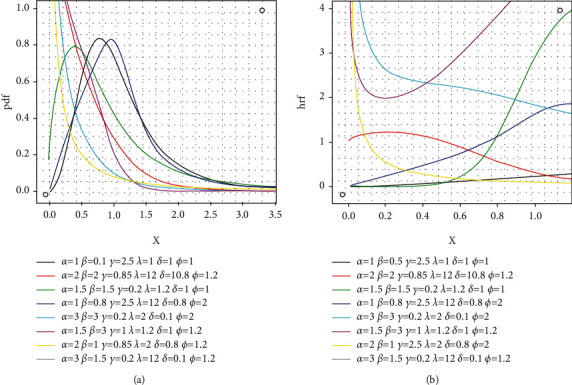
Selected *f*(*x*) and *h*(*x*) graphs for EGIKw-Burr XII model.

A rv*X* with the above pdf is denoted as *X* ~ EGIKw − BurrXII(*α*, *β*, *γ*, *λ*, *ψ*, *ξ*).  Figure 4 displays some interesting shapes of EGIKw-Burr XII pdf and hrf. It is obvious from these plots that great flexibility is achieved with the proposed models.

## 4. Structural Properties of EGIKw-G Family of Distributions

In this part of article, we provide some useful expressions for EGIKw-G class including explicit expansions of density and cumulative distribution function, *r*^th^ moment, *m*  *d*, moment generating function (mgf), and pdf of order statistics.

### 4.1. Expansions for EGIKw-G cdf and pdf

We express EGIKw-G cdf and pdf in terms of finite (or infinite) weighted sums of exponentiated-G cdf and pdf, respectively. Consider the EGIKw-G cdf given by (4)

(18) $$\begin{array}{c} F_{\text{EGIKw}-\text{G}}\left(x\right)={\left[1-{\left[1+{\left(\frac{W^{\lambda }\left(x\right)}{1-W^{\lambda }\left(x\right)}\right)}^{\gamma }\right]}^{-\alpha -1}\right]}^{\beta }. \end{array}$$

For *d* > 0 real noninteger and |*y*| < 1, the power series representations are

(19) $$\begin{array}{c} {\left(1-y\right)}^{d}=\overset{\infty }{\underset{i=0}{\sum }}\left(\begin{array}{c} d\\ i \end{array}\right){\left(-1\right)}^{i}y^{i}, \end{array}$$

(20) $$\begin{array}{c} {\left(1+y\right)}^{-d}=\overset{\infty }{\underset{i=0}{\sum }}\left(\begin{array}{c} d+i-1\\ i \end{array}\right){\left(-1\right)}^{i}y^{i}. \end{array}$$

For  *d* > 0 integer value,

(21) $$\begin{array}{c} {\left(1-y\right)}^{d}=\sum_{i=0}^{\infty }\left(\begin{array}{c} d\\ i \end{array}\right){\left(-1\right)}^{i}y^{i}. \end{array}$$

Using the series expansions given above, the EGIKw-G distribution function (4) is rewritten as

(22) $$\begin{array}{c} F_{\text{EGIKw}-\text{G}}\left(x\right)=\overset{\infty }{\underset{i=0}{\sum }}{\left(-1\right)}^{i}\left(\begin{array}{c} \beta \\ i \end{array}\right){\left[1+{\left(\frac{W^{\lambda }\left(x\right)}{1-W^{\lambda }\left(x\right)}\right)}^{\gamma }\right]}^{-\alpha i}\\ =\overset{\infty }{\underset{i,j=0}{\sum }}{\left(-1\right)}^{i+j}\left(\begin{array}{c} \beta \\ i \end{array}\right)\left(\begin{array}{c} \alpha i+j-1\\ j \end{array}\right){\left(\frac{W^{\lambda }\left(x\right)}{1-W^{\lambda }\left(x\right)}\right)}^{\gamma j}\\ =\overset{\infty }{\underset{i,j,k=0}{\sum }}{\left(-1\right)}^{i+j}\left(\begin{array}{c} \beta \\ i \end{array}\right)\left(\begin{array}{c} \alpha i+j-1\\ j \end{array}\right)\left(\begin{array}{c} \gamma j+k-1\\ k \end{array}\right)W{\left(x\right)}^{\lambda \left(\gamma j+k\right)}\\ =\overset{\infty }{\underset{i,j,k=0}{\sum }}\iota_{i,j,k}W{\left(x\right)}^{\lambda \left(\gamma j+k\right)}, \end{array}$$

where $\iota_{i,j,k}={\left(-1\right)}^{i+j}\left(\begin{array}{c} \beta \\ i \end{array}\right)\left(\begin{array}{c} \alpha i+j-1\\ j \end{array}\right)\left(\begin{array}{c} \gamma j+k-1\\ k \end{array}\right)$. For any integer value of *β*, index *i* is stopped at *β*, *β*; for an integer *α*, the index *j* stops at *αi*+*j* − 1, *αi*+*j* − 1; and for an integer value of *γ*, index *k* is stopped at *γj*+*k* − 1, *γj*+*k* − 1. Thus, (22) reveals that EGIKw-G pdf can be written in baseline pdf as a multiple of its cdf's power series. Otherwise, in case of *γ* to be a real noninteger, the *W*(*x*)^*λ*(*γj*+*k*)^ in (22) can have following form

(23) $$\begin{array}{c} W{\left(x\right)}^{\lambda \left(\gamma j+k\right)}={\left[1-\left[1-W\left(x\right)\right]\right]}^{\lambda \left(\gamma j+k\right)}=\overset{\infty }{\underset{l=0}{\sum }}{\left(-1\right)}^{l}\left(\begin{array}{c} \lambda \left(\gamma j+k\right)\\ l \end{array}\right){\left[1-W\left(x\right)\right]}^{l}. \end{array}$$

Using the binomial expansion for [1 − *W*(*x*)]^*l*^, we obtain

(24) $$\begin{array}{c} {\left[1-W\left(x\right)\right]}^{l}=\sum_{r=0}^{l}{\left(-1\right)}^{r}\left(\begin{array}{c} l\\ r \end{array}\right)W{\left(x\right)}^{r}. \end{array}$$

Using (24) into (23), we have

(25) $$\begin{array}{c} W{\left(x\right)}^{\lambda \left(\gamma j+k\right)}=\overset{\infty }{\underset{l=0}{\sum }}\sum_{r=0}^{l}{\left(-1\right)}^{l+r}\left(\begin{array}{c} \lambda \left(\gamma j+k\right)\\ l \end{array}\right)\left(\begin{array}{c} l\\ r \end{array}\right)W{\left(x\right)}^{r}. \end{array}$$

Further, (4) is rewritten as

(26) $$\begin{array}{c} F_{\mathrm{EGIKw-G}}\left(x\right)=\overset{\infty }{\underset{i,j,k,l=0}{\sum }}\sum_{r=0}^{l}t_{i,j,k,l,r}W{\left(x\right)}^{r}, \end{array}$$

where $t_{i,j,k,l,r}={\left(-1\right)}^{l+r}\left(\begin{array}{c} \lambda \left(\gamma j+k\right)\\ l \end{array}\right)\left(\begin{array}{c} l\\ r \end{array}\right)\iota_{i,j,k}$. Replacing ∑_*l*=0_^*∞*^∑_*r*=0_^*l*^ by ∑_*r*=0_^*∞*^∑_*l*=*r*_^*∞*^ in (22), we have

(27) $$\begin{array}{c} F_{\mathrm{EGIKw-G}}\left(x\right)=\sum_{r=0}^{\infty }z_{r}W{\left(x\right)}^{r}, \end{array}$$

where *z*_*r*_=∑_*i*,*j*,*k*=0_^*∞*^∑_*l*=*r*_^*∞*^*t*_*i*,*j*,*k*,*l*,*r*_ is sum in constants. The expansion (27) holds for all real noninteger *γ* values. It should be noted that EGIKw-G cdf can also be provided in the form of exponential-G cdf as

(28) $$\begin{array}{c} F_{\mathrm{EGIKw-G}}\left(x\right)=\sum_{r=0}^{\infty }z_{r}V_{r}\left(x\right), \end{array}$$

where *V*_*r*_(*x*)=*W*(*x*)^*r*^ denotes exponential-*G*cdf, where *r* is power parameter. The corresponding results for EGIKw-G pdf are obtained by differentiating (22) for *γ* > 0 integer and by (27) and (28) for *γ* > 0 real noninteger value, respectively, as

(29) $$\begin{array}{c} f_{\mathrm{EGIKw-G}}\left(x\right)=w\left(x\right)\overset{\infty }{\underset{i,j,k=0}{\sum }}l_{i,j,k}^{\prime \prime }W{\left(x\right)}^{\lambda \left(\gamma j+k\right)-1}, \end{array}$$

(30) $$\begin{array}{c} f_{\mathrm{EGIKw-G}}\left(x\right)=w\left(x\right)\overset{\infty }{\underset{r=0}{\sum }}{\overset{⌣}{z}}_{r}W{\left(x\right)}^{r}, \end{array}$$

(31) $$\begin{array}{c} f_{\text{EGIKw}-\text{G}}\left(x\right)=\sum_{r=0}^{\infty }z_{r}^{\prime \prime }v_{r+1}\left(x\right), \end{array}$$

where $l_{i,j,k}^{\prime \prime }=\lambda \left(\gamma j+k\right)\iota_{i,j,k},\;{\overset{⌣}{z}}_{r}=\left(r+1\right)z_{r+1},\;z_{r}^{\prime \prime }=z_{r+1}$ for *r*=0,1,2,…, *r*=0,1,2,…; *v*_*r*+1_(*x*)=(*r*+1)*w*(*x*)*W*(*x*)^*r*^ is exponential-*G* density function having parameter (*r*+1). Equation (31) expresses EGIKw-G density in terms of exponential-*G* densities. Equations (29)–(31) are among main results from this section.

### 4.2. Moments

Moments play a crucial role in studying some important characteristics (tendency, dispersion, skewness, kurtosis, etc.) of a distribution. The *p*^th^ EGIKw-G moment can be given as weighted sum in probability weighted moments (PWMs) of order (*p*, *q*) of the parent distribution. Let *X* and *Y*, respectively, come from EGIKw-G and baseline *G* distribution. We can write *p*^th^ raw moment for *X* in terms of (*p*, *q*)^th^ PWM (*τ*_*p*,*q*_=*E*[*Y*^*p*^*G*(*Y*)^*q*^]=∫*x*^*p*^*w*(*x*)*W*(*x*)^*q*^d*x*, (*q*=0,1,…)) of *Y*. For *γ* > 0 integer, we have

(32) $$\begin{array}{c} \mu_{p}^{\prime }=E\left(X^{p}\right)=\sum_{i,j,k=0}^{\infty }l_{i,j,k}^{\prime \prime }\tau_{p,\lambda \left(\gamma j+k\right)-1}, \end{array}$$

where *τ*_*p*,*λ*(*γj*+*k*)−1_, is the (*p*, *λ*(*γj*+*k*) − 1)^th^ PWM of baseline distribution and *l*_*i*,*j*,*k*_^″^ is defined in (29). For *γ* > 0 noninteger, we can write

(33) $$\begin{array}{c} \mu_{p}^{\prime }=E\left(X^{p}\right)=\sum_{r=0}^{\infty }{\overset{⌣}{z}}_{r}\tau_{p,r}, \end{array}$$

where ${\overset{⌣}{z}}_{r}$ is from (30) and *τ*_*p*,*r*_ denotes (*p*, *r*)^th^ PWM of baseline distribution. Hence, moments for any EGIKw-G model can be calculated using baseline PWMs.

Furthermore, *μ*_*p*_′ can be obtained using baseline qf, *Q*(*u*)=*W*^−1^(*u*)=*x*. For *γ* > 0 integer, from (22), and for *γ* > 0 noninteger, from (30), we, respectively, obtain

(34) $$\begin{array}{c} \mu_{p}^{\prime }=\overset{\infty }{\underset{i,j,k=0}{\sum }}l_{i,j,k}^{\prime \prime }\int x^{p}w\left(x\right)W{\left(x\right)}^{\lambda \left(\gamma j+k\right)-1}dx,\\ \mu_{p}^{\prime }=\overset{\infty }{\underset{r=0}{\sum }}{\overset{⌣}{z}}_{r}\int x^{p}w\left(x\right)W{\left(x\right)}^{r}\text{d}x. \end{array}$$

Using *u*=*W*(*x*_*u*_) in the above expressions, we have

(35) $$\begin{array}{c} \mu_{p}^{\prime }=\overset{\infty }{\underset{i,j,k=0}{\sum }}l_{i,j,k}^{\prime \prime }\int_{0}^{1}u^{\lambda \left(\gamma j+k\right)-1}Q{\left(u\right)}^{p}\text{d}u,\\ \mu_{p}^{\prime }=\overset{\infty }{\underset{r=0}{\sum }}{\overset{⌣}{z}}_{r}\int_{0}^{1}u^{r}Q{\left(u\right)}^{p}\text{d}u. \end{array}$$

respectively. Moreover, we can also provide the EGIKw-G moments in the form of exponential-*G* moments. Let *X*_*r*+1_ be an exponential-*G*rv with cdf, *V*_*r*+1_(*x*)=*W*(*x*)^*r*^, and pdf, *v*_*r*+1_(*x*)=(*r*+1)*w*(*x*)*W*(*x*)^*r*^, and (*r* + 1) be the power parameter, so

(36) $$\begin{array}{c} E\left(X_{r+1}^{p}\right)=\int x^{p}v_{r+1}\left(x\right)\text{d}x. \end{array}$$

Hence, we have

(37) $$\begin{array}{c} \mu_{p}^{\prime }=\sum_{r=0}^{\infty }z_{r}^{\prime \prime }\int x^{p}v_{r+1}\left(x\right)dx, \end{array}$$

where *z*_*r*_^″^ is defined in (31). Thus, EGIKw-G moments can be written as function of baseline exponential-*G* moments.

### 4.3. Moment Generating Function

Let *X* ∼ EGIKw-G (*α*, *β*, *γ*, *λ*). We consider various expressions of mgf for *X* as

(38) $$\begin{array}{c} M\left(t\right)=E\left[\exp\left(tX\right)\right]\\ =E\left[\overset{\infty }{\underset{p=0}{\sum }}\frac{X^{p}}{p!}t^{p}\right]\\ =\overset{\infty }{\underset{p=0}{\sum }}\left(\frac{\mu_{p}^{\prime }}{p!}\right)t^{p}, \end{array}$$

where *μ*_*p*_′=*E*(*X*^*p*^) is the *p*^th^ EGIKw-G noncentral moment. Another representation of *M*(*t*), when *γ* > 0 integer, is derived from (29) as

(39) $$\begin{array}{c} M\left(t\right)=\sum_{i,j,k=0}^{\infty }l_{i,j,k}^{\prime \prime }\varphi \left(t,\lambda \left(\gamma j+k\right)-1\right), \end{array}$$

where the function *φ*(*t*, *λ*(*γj*+*k*) − 1)=∫exp(*tx*)*w*(*x*)*W*(*x*)^*λ*(*γj*+*k*)−1^d*x* is obtained using baseline qf as

(40) $$\begin{array}{c} \varphi \left(t,\lambda \left(\gamma j+k\right)-1\right)=\int_{0}^{1}u^{\lambda \left(\gamma j+k\right)-1}\exp\left(tQ\left(u\right)\right)\text{d}u. \end{array}$$

For *γ* > 0 noninteger, using (30) we also have

(41) $$\begin{array}{c} M\left(t\right)=\sum_{r=0}^{\infty }{\overset{⌣}{z}}_{r}\varphi \left(t,r\right)\text{,} \end{array}$$

and the function *φ*(*t*, *r*)=∫exp(*tx*)*w*(*x*)*W*(*x*)^*r*^d*x* is easily deduced from baseline qf as

(42) $$\begin{array}{c} \varphi \left(t,r\right)=\int_{0}^{1}u^{r}\exp\left(tQ\left(u\right)\right)\text{d}u. \end{array}$$

Another representation for *M*(*t*) for *γ* > 0 noninteger is obtained from (31) as

(43) $$\begin{array}{c} M\left(t\right)=\sum_{r=0}^{\infty }z_{r}^{\prime \prime }M_{r+1}\left(t\right), \end{array}$$

where *M*_*r*+1_(*t*) is mgf of *X*∼ exponential-*G(r+1)*rv. Hence, *M*(*t*) of any EGIKw-G model can be determined from the corresponding exponential-G mgf.

### 4.4. Mean Deviations

The *m*  *d* of a population measures its amount of scattering. For a rv*X* having pdf*f(x)* and cdf*F(x)*, the md about mean and md about median are, respectively, written as *δ*_*μ*_(*X*) and *δ*_*M*_(*X*) and are, respectively, given by

(44) $$\begin{array}{c} \delta_{\mu }\left(X\right)=E\left(\left|X-\mu_{1}^{\prime }\right|\right)\\ =2\mu_{1}^{\prime }F\left(\mu_{1}^{\prime }\right)-2T\left(\mu_{1}^{\prime }\right),\\ \delta_{M}\left(X\right)=E\left(\left|X-M\right|\right)\\ =\mu_{1}^{\prime }-2T\left(M\right), \end{array}$$

where *μ*_1_′ is the first ordinary moment, *F*(*μ*_1_′) is from (4), *M* is median obtained from (7) for *u*=(1/2), and *T*(*z*)=∫_−*∞*_^*z*^*xf*(*x*)d*x* represents 1^st^ incomplete moment. Using parent qf, two additional expressions for *T*(*x*) are derived. Firstly, when *γ* > 0 integer,

(45) $$\begin{array}{c} T\left(z\right)=\sum_{i,j,k=0}^{\infty }l_{i,j,k}^{\prime \prime }\int_{0}^{G\left(z\right)}u^{\lambda \left(\gamma j+k\right)-1}Q\left(u\right)\text{d}u\text{.} \end{array}$$

For *γ* > 0 real noninteger, we have

(46) $$\begin{array}{c} T\left(z\right)=\sum_{r=0}^{\infty }{\overset{⌣}{z}}_{r}\int_{0}^{G\left(z\right)}u^{r}Q\left(u\right)\text{d}u, \end{array}$$

where $l_{i,j,k}^{\prime \prime },\;{\overset{⌣}{z}}_{r}$ are defined in (29) and (30), respectively. Another useful expression for *T*(*z*) is obtained from exponential-*G* distribution as

(47) $$\begin{array}{c} T\left(z\right)=\sum_{r=0}^{\infty }z_{r}^{\prime \prime }\int_{-\infty }^{z}xv_{r+1}\left(x\right)\text{d}x, \end{array}$$

where *z*_*r*_^″^ is defined by (31).

### 4.5. Rényi Entropy

Entropies of any rv, say *X*, are measures of diversity of uncertainty. These measures have been used in various fields including engineering, physics, and economics. Rényi entropy is the most popular measure of entropy and is given as (Rényi [27])

(48) $$\begin{array}{c} I_{\zeta }\left(x\right)=\frac{1}{1-\zeta }\log\left[\int_{-\infty }^{\infty }f^{\zeta }\left(x\right)\text{d}x\right],\;\zeta >0,\;\zeta \neq 1. \end{array}$$

Using (19)–(21) the pdf *f*_EGIKw−G_^*ζ*^(*x*) becomes

(49) $$\begin{array}{c} f_{\text{EGIKw}-\text{G}}^{\zeta }\left(x\right)={\left(\alpha \beta \gamma \lambda \right)}^{\zeta }w^{\zeta }\left(x\right)\overset{\infty }{\underset{i,j,k=0}{\sum }}{\tilde{l}}_{i,j,k}W{\left(x\right)}^{\lambda \left(\gamma j+k\right)+\zeta \left(\gamma \lambda -1\right)}. \end{array}$$

Hence,

(50) $$\begin{array}{c} I_{\zeta }\left(x\right)=\frac{1}{1-\zeta }\log\left[{\left(\alpha \beta \gamma \lambda \right)}^{\zeta }\overset{\infty }{\underset{i,j,k=0}{\sum }}{\tilde{l}}_{i,j,k}\int_{-\infty }^{\infty }W{\left(x\right)}^{\lambda \left(\gamma j+k\right)+\zeta \left(\gamma \lambda -1\right)}w^{\zeta }\left(x\right)\text{d}x\right]. \end{array}$$

Equivalently depending on the parent qf,

(51) $$\begin{array}{c} I_{\zeta }\left(x\right)=\frac{1}{1-\zeta }\log\left[{\left(\alpha \beta \gamma \lambda \right)}^{\zeta }\overset{\infty }{\underset{i,j,k=0}{\sum }}{\tilde{l}}_{i,j,k}\int_{0}^{1}u^{\lambda \left(\gamma j+k\right)+\zeta \left(\gamma \lambda -1\right)}w^{\zeta -1}\left(Q\left(u\right)\right)\text{d}x\right], \end{array}$$

where ${\tilde{l}}_{i,j,k}={\left(-1\right)}^{i+j}\left(\begin{array}{c} \zeta \left(\beta -1\right)\\ i \end{array}\right)\left(\begin{array}{c} \alpha i+\zeta \left(\alpha +1\right)+j-1\\ j \end{array}\right)\left(\begin{array}{c} \gamma j+\zeta \left(\gamma +1\right)+k-1\\ k \end{array}\right)$. In this section, (50) and (51) are main results.

### 4.6. Stress-Strength Reliability

The reliability measure of industrial components has crucial role especially in engineering. The reliability of a product is the probability that it will do its intended job up to a specific time, given that it is operating under normal conditions. The component fails when *X*_2_ (random stress) placed on it exceeds *X*_1_ (random strength), and for *X*_1_ > *X*_2_ it will work satisfactorily. Thus, *R*=*P*(*X*_2_ < *X*_1_) measures the component's reliability (Kotz et al. [28]). Let X_1_ and X_2_ be independent rv, rv; let *X*_1_ be an EGIKw-G *rv* with *f*_1_(*x*), (5), and parameters *α*_1_, *β*_1_, *γ*_1_, *λ*_1_; and let *X*_2_ be a *rv* with cdf *F*_2_(*x*), (4), and parameters *α*_2_, *β*_2_, *γ*_2_, *λ*_2_ with common baseline parametric space *ϑ*. Then, *R* is obtained as

(52) $$\begin{array}{c} R=\int f_{1}\left(x\right)F_{2}\left(x\right)\text{d}x\\ =\alpha_{1}\beta_{1}\gamma_{1}\lambda_{1}\int w\left(x,\vartheta \right)W^{\gamma_{1}\lambda_{1}-1}\left(x,\vartheta \right){\left[1-W^{\lambda_{1}}\left(x,\vartheta \right)\right]}^{-\gamma_{1}-1}\\ \times {\left[1+{\left(\frac{W^{\lambda_{1}}\left(x,\vartheta \right)}{1-W^{\lambda_{1}}\left(x,\vartheta \right)}\right)}^{\gamma_{1}}\right]}^{-\alpha_{1}-1}{\left[1-{\left[1+{\left(\frac{W^{\lambda_{1}}\left(x,\vartheta \right)}{1-W^{\lambda_{1}}\left(x,\vartheta \right)}\right)}^{\gamma_{1}}\right]}^{-\alpha_{1}}\right]}^{\beta_{1}-1}\\ \times {\left[1-{\left[1+{\left(\frac{W^{\lambda_{2}}\left(x,\vartheta \right)}{1-W^{\lambda_{2}}\left(x,\vartheta \right)}\right)}^{\gamma_{2}}\right]}^{-\alpha_{2}}\right]}^{\beta_{2}}\text{d}x. \end{array}$$

Alternatively, with the change of rv, *X*=*Q*_1_(*u*),

(53) $$\begin{array}{c} R=\int_{0}^{1}F_{2}\left(Q_{1}\left(u\right)\right)\text{d}u\\ =\int_{0}^{1}{\left\{1-{\left[1+{\left\{{\left[1+{\left\{{\left(1-u^{\frac{1}{\beta_{1}}}\right)}^{-\frac{1}{\alpha_{1}}}-1\right\}}^{-\frac{1}{\gamma_{1}}}\right]}^{\frac{\lambda_{2}}{\lambda_{1}}}-1\right\}}^{-\gamma_{2}}\right]}^{-\alpha_{2}}\right\}}^{\beta_{2}}\text{d}u, \end{array}$$

where *Q*_1_(*u*) is qf from (7) corresponding to *f*_1_(*x*). Interestingly, we see that *R* is independent of *W(x)*, the baseline distribution. Additionally, various different forms will be yielded by using linear expression. One form is derived for *γ*_1_, *γ*_2_ > 0 integers by using

(54) $$\begin{array}{c} f_{1}\left(x\right)=w\left(x\right)\overset{\infty }{\underset{t,u,v=0}{\sum }}l_{t,u,v}^{\prime \prime }W{\left(x\right)}^{\lambda_{1}\left(\gamma_{1}u+v\right)-1},\\ F_{2}\left(x\right)=\overset{\infty }{\underset{i,j,k=0}{\sum }}\iota_{i,j,k}W{\left(x\right)}^{\lambda_{2}\left(\gamma_{2}j+k\right)}, \end{array}$$

where $l_{t,u,v}=\lambda_{1}\left(\gamma_{1}u+v\right){\left(-1\right)}^{t+u}\left(\begin{array}{c} \beta_{1}\\ t \end{array}\right)\left(\begin{array}{c} \alpha_{1}t+u-1\\ u \end{array}\right)\left(\begin{array}{c} \gamma_{1}u+v-1\\ v \end{array}\right)$, $\text{and }\iota_{i,j,k}={\left(-1\right)}^{i+j}\left(\begin{array}{c} \beta_{2}\\ i \end{array}\right)\left(\begin{array}{c} \alpha_{2}i+j-1\\ j \end{array}\right)\left(\begin{array}{c} \gamma_{2}j+k-1\\ k \end{array}\right)$. Thus,

(55) $$\begin{array}{c} R=\overset{\infty }{\underset{i,j,k,t,u,v=0}{\sum }}\iota_{i,j,k}l_{t,u,v}^{\prime \prime }\int_{-\infty }^{\infty }w\left(x\right)W{\left(x\right)}^{\lambda_{1}\left(\gamma_{1}u+v\right)+\lambda_{2}\left(\gamma_{2}j+k\right)-1}\text{d}x\\ =\overset{\infty }{\underset{i,j,k,t,u,v=0}{\sum }}\frac{\iota_{i,j,k}l_{t,u,v}^{\prime \prime }}{\lambda_{1}\left(\gamma_{1}u+v\right)+\lambda_{2}\left(\gamma_{2}j+k\right)}. \end{array}$$

Similar expressions can be obtained for the case *γ*_1_, *γ*_2_ > 0 nonintegers. As usual, when *α*_1_=*α*_2_,  *β*_1_=*β*_2_,  *γ*_1_=*γ*_2_,  *λ*_1_=*λ*_2_, i.e., corresponding to the identically distributed case, we have *R*=(1/2).

### 4.7. Lorenz *L*(*p*) and Bonferroni *B*(*p*) Curves

The Lorenz curve for *γ* > 0 integer, is given as follows:

(56) $$\begin{array}{c} L\left(p\right)=\frac{E_{X\leq x}}{E\left(X\right)}\\ =\frac{1}{E\left(X\right)}\int_{0}^{x}tf\left(t\right)\text{d}t\\ =\frac{1}{\mu }\overset{\infty }{\underset{i,j,k=0}{\sum }}l_{t,u,v}^{\prime \prime }\int_{0}^{x}tW{\left(t\right)}^{\lambda \left(\gamma j+k\right)-1}w\left(t\right)\text{d}t. \end{array}$$

Equivalently based on parent qf and in the form of exponential-*G* distribution, we have

(57) $$\begin{array}{c} L\left(p\right)=\frac{1}{\mu }\overset{\infty }{\underset{i,j,k=0}{\sum }}l_{i,j,k}^{\prime \prime }\int_{0}^{W\left(x\right)}u^{\lambda \left(\gamma j+k\right)-1}Q\left(u\right)\text{d}u,\\ L\left(p\right)=\frac{1}{\mu }\overset{\infty }{\underset{i,j,k=0}{\sum }}\frac{l_{i,j,k}^{\prime \prime }}{\lambda \left(\gamma j+k\right)}\int_{0}^{x}tv_{\lambda \left(\gamma j+k\right)}\left(t\right)\text{d}t, \end{array}$$

respectively. The corresponding expressions for Bonferroni curve are, respectively, given by (58)–(60) as

(58) $$\begin{array}{c} B\left(p\right)=\frac{E_{X\leq x}}{F\left(X\right)E\left(X\right)}\\ =\frac{1}{F\left(X\right)E\left(X\right)}\int_{0}^{x}tf\left(t\right)\text{d}t\\ =\frac{1}{\mu F\left(X\right)}\overset{\infty }{\underset{i,j,k=0}{\sum }}l_{i,j,k}^{\prime \prime }\int_{0}^{x}tW{\left(t\right)}^{\lambda \left(\gamma j+k\right)-1}w\left(t\right)\text{d}t, \end{array}$$

(59) $$\begin{array}{c} B\left(p\right)=\frac{1}{\mu F\left(X\right)}\overset{\infty }{\underset{i,j,k=0}{\sum }}l_{i,j,k}^{\prime \prime }\int_{0}^{W\left(x\right)}u^{\lambda \left(\gamma j+k\right)-1}Q\left(u\right)\text{d}u, \end{array}$$

(60) $$\begin{array}{c} B\left(p\right)=\frac{1}{\mu F\left(X\right)}\overset{\infty }{\underset{i,j,k=0}{\sum }}\frac{l_{i,j,k}^{\prime \prime }}{\lambda \left(\gamma j+k\right)}\int_{0}^{x}tv_{\lambda \left(\gamma j+k\right)}\left(t\right)\text{d}t. \end{array}$$

Similar expressions can be obtained using (30) for the case of *γ* > 0 noninteger.

### 4.8. Moments of Residual Life Function

In reliability theory and life testing problems, residual life has an important role. The *n*^th^ moment is provided by

(61) $$\begin{array}{c} m_{n}\left(t\right)=E\left[\frac{{\left(X-t\right)}^{n}}{X>t}\right]\\ =\frac{1}{R\left(t\right)}\int_{t}^{\infty }{\left(x-t\right)}^{n}f\left(x\right)\text{d}x\\ =\frac{1}{R\left(t\right)}\overset{n}{\underset{a=0}{\sum }}\left(\begin{array}{c} n\\ a \end{array}\right){\left(-t\right)}^{n-a}\int_{t}^{\infty }x^{a}f\left(x\right)\text{d}x. \end{array}$$

Similarly, *n*^th^ residual moment of a *rv* having EGIKw-G distribution for *γ* > 0 integer and for *γ* > 0 noninteger is obtained by inserting pdf of (29) and (30) in the above expression, respectively, as

(62) $$\begin{array}{c} m_{n}\left(t\right)=\frac{1}{R\left(t\right)}\overset{\infty }{\underset{i,j,k=0}{\sum }}\overset{n}{\underset{a=0}{\sum }}l_{i,j,k}^{\prime \prime }\left(\begin{array}{c} n\\ a \end{array}\right){\left(-t\right)}^{n-a}\int_{t}^{\infty }x^{a}W{\left(x\right)}^{\lambda \left(\gamma j+k\right)-1}w\left(x\right)\text{d}x,\\ m_{n}\left(t\right)=\frac{1}{R\left(t\right)}\overset{\infty }{\underset{r=0}{\sum }}\overset{n}{\underset{a=0}{\sum }}{\overset{⌣}{z}}_{r}\left(\begin{array}{c} n\\ a \end{array}\right){\left(-t\right)}^{n-a}\int_{t}^{\infty }x^{a}W{\left(x\right)}^{r}w\left(x\right)\text{d}x. \end{array}$$

Equivalently depending upon the parent qf, we have

(63) $$\begin{array}{c} m_{n}\left(t\right)=\frac{1}{R\left(t\right)}\overset{\infty }{\underset{i,j,k=0}{\sum }}\overset{n}{\underset{a=0}{\sum }}l_{i,j,k}^{\prime \prime }\left(\begin{array}{c} n\\ a \end{array}\right){\left(-t\right)}^{n-a}\int_{G\left(t\right)}^{1}u^{\lambda \left(\gamma j+k\right)-1}Q{\left(u\right)}^{a}\text{d}u,\\ m_{n}\left(t\right)=\frac{1}{R\left(t\right)}\overset{\infty }{\underset{r=0}{\sum }}\overset{n}{\underset{a=0}{\sum }}{\overset{⌣}{z}}_{r}\left(\begin{array}{c} n\\ a \end{array}\right){\left(-t\right)}^{n-a}\int_{G\left(t\right)}^{1}u^{r}Q{\left(u\right)}^{a}\text{d}u. \end{array}$$

An alternative representation can be derived from exponential-G distribution as

(64) $$\begin{array}{c} m_{n}\left(t\right)=\frac{1}{R\left(t\right)}\overset{\infty }{\underset{r=0}{\sum }}\overset{n}{\underset{a=0}{\sum }}l_{r}^{\prime \prime }\left(\begin{array}{c} n\\ a \end{array}\right){\left(-t\right)}^{n-a}\int_{t}^{\infty }x^{a}v_{r+1}\left(x\right)\text{d}x. \end{array}$$

### 4.9. Order Statistics

Order statistics are useful in detection of outliers and robust statistical estimation, characterization of probability distributions, reliability analysis, analysis of censored samples, etc. Let *X*_1_, *X*_2_,…, *X*_*n*_ be *n* *rv* from the EGIKw-G distribution. Let *X*_(1)_, *X*_(2)_,…, *X*_(*n*)_ denote the order statistics. The density of *i*^th^ ordered value is

(65) $$\begin{array}{c} f_{i:n}\left(x\right)=\frac{f\left(x\right)}{B\left(i,n-i+1\right)}F{\left(x\right)}^{i-1}{\left[1-F\left(x\right)\right]}^{n-i}\\ =\frac{f\left(x\right)}{B\left(i,n-i+1\right)}\overset{n-i}{\underset{h=0}{\sum }}{\left(-1\right)}^{h}\left(\begin{array}{c} n-i\\ h \end{array}\right)F{\left(x\right)}^{h+i-1}, \end{array}$$

where *B*(., .) is expression for beta function. We offer the pdf of EGIKw-G order statistics in the form of baseline pdf as multiple of *W*(*x*). Replacing (27) in the above expression yields

(66) $$\begin{array}{c} F{\left(x\right)}^{h+i-1}={\left[\overset{\infty }{\underset{t=0}{\sum }}z_{t}W{\left(x\right)}^{t}\right]}^{h+i-1}\\ ={\left[\overset{\infty }{\underset{t=0}{\sum }}z_{t}u^{t}\right]}^{h+i-1}. \end{array}$$

Let us consider

(67) $$\begin{array}{c} {\left(\overset{\infty }{\underset{t=0}{\sum }}s_{t}y^{t}\right)}^{z}=\overset{\infty }{\underset{t=0}{\sum }}c_{t,z}y^{t}, \end{array}$$

where *c*_0,*z*_=(*s*_0_)^*z*^,  *c*_*t*,*z*_=(*ts*_0_)^−1^∑_*m*=1_^*t*^[*m*(*z*+1) − *t*]*s*_*m*_*c*_*t*−*m*,*z*_ (Gradshteyn and Ryzhik [1]). Hence, we have

(68) $$\begin{array}{c} F{\left(x\right)}^{h+i-1}=\overset{\infty }{\underset{t=0}{\sum }}c_{t,h+i-1}W{\left(x\right)}^{t}\\ =\overset{\infty }{\underset{t=0}{\sum }}c_{t,h+i-1}u^{t}\text{,} \end{array}$$

with *c*_0,*h*+*i*−1_=(*z*_0_)^*h*+*i*−1^,  *c*_*t*,*h*+*i*−1_=(*tz*_0_)^−1^∑_*m*=1_^*t*^[*m*(*h*+*i*) − *t*]*z*_*m*_*c*_*t*−*m*,*h*+*i*−1_. Using (68) in (65) with (29) for *γ* > 0 integer and with (30) for *γ* > 0 noninteger, we, respectively, obtain

(69) $$\begin{array}{c} f_{i:n}\left(x\right)=\frac{w\left(x\right)}{B\left(i,n-i+1\right)}\overset{\infty }{\underset{l,j,k,t=0}{\sum }}\overset{n-i}{\underset{h=0}{\sum }}l_{i,j,k}^{\prime \prime }c_{t,h+i-1}{\left(-1\right)}^{h}\left(\begin{array}{c} n-i\\ h \end{array}\right)W{\left(x\right)}^{\lambda \left(\gamma j+k\right)+t-1},\\ f_{i:n}\left(x\right)=\frac{w\left(x\right)}{B\left(i,n-i+1\right)}\overset{\infty }{\underset{r,t=0}{\sum }}\overset{n-i}{\underset{h=0}{\sum }}{\overset{⌣}{z}}_{r}c_{t,h+i-1}{\left(-1\right)}^{h}\left(\begin{array}{c} n-i\\ h \end{array}\right)W{\left(x\right)}^{r+t}. \end{array}$$

Clearly, the above equations can be given in the form of exponential-G densities as

(70) $$\begin{array}{c} f_{i:n}\left(x\right)=\overset{\infty }{\underset{l,j,k,t=0}{\sum }}\overset{n-i}{\underset{h=0}{\sum }}\frac{w_{i,j,k}^{\prime \prime }c_{t,h+i-1}{\left(-1\right)}^{h}\left(\begin{array}{c} n-i\\ h \end{array}\right)}{B\left(i,n-i+1\right)\left(\lambda \left(\gamma j+k\right)+t\right)}\;v_{\lambda \left(\gamma j+k\right)+t}\left(x\right). \end{array}$$

(71) $$\begin{array}{c} f_{i:n}\left(x\right)=\overset{\infty }{\underset{r,t=0}{\sum }}\overset{n-i}{\underset{h=0}{\sum }}\frac{{\overset{⌣}{z}}_{r}c_{t,h+i-1}{\left(-1\right)}^{h}\left(\begin{array}{c} n-i\\ h \end{array}\right)}{B\left(i,n-i+1\right)\left(r+t+1\right)}v_{r+t+1}\left(x\right). \end{array}$$

Equations (70) for *γ* > 0 integer and (71) for *γ* > 0 noninteger immediately yield the pdf of EGIKw-G order statistics as a function of exponential-G pdf,s. Hence, the corresponding moments can be provided in the form of baseline PWMs for *γ* > 0 integer and for *γ* > 0 noninteger, respectively, by

(72) $$\begin{array}{c} E_{i:n}\left(x^{s}\right)=\frac{1}{B\left(i,n-i+1\right)}\overset{\infty }{\underset{l,j,k,t=0}{\sum }}\overset{n-i}{\underset{h=0}{\sum }}w_{i,j,k}^{\prime \prime }c_{t,h+i-1}{\left(-1\right)}^{h}\left(\begin{array}{c} n-i\\ h \end{array}\right)\tau_{s,\lambda \left(\gamma j+k\right)+t-1},\\ E_{i:n}\left(x^{s}\right)=\frac{1}{B\left(i,n-i+1\right)}\overset{\infty }{\underset{r,t=0}{\sum }}\overset{n-i}{\underset{h=0}{\sum }}{\overset{⌣}{z}}_{r}c_{t,h+i-1}{\left(-1\right)}^{h}\left(\begin{array}{c} n-i\\ h \end{array}\right)\tau_{s,r+t}. \end{array}$$

Depending upon the parent qf for *γ* > 0 integer and for *γ* > 0 noninteger, we, respectively, obtain

(73) $$\begin{array}{c} E_{i:n}\left(x^{s}\right)=\overset{\infty }{\underset{l,j,k,t=0}{\sum }}\overset{n-i}{\underset{h=0}{\sum }}\frac{w_{i,j,k}^{\prime \prime }c_{t,h+i-1}{\left(-1\right)}^{h}\left(\begin{array}{c} n-i\\ h \end{array}\right)}{B\left(i,n-i+1\right)}\int_{0}^{1}u^{\lambda \left(\gamma j+k\right)+t-1}Q{\left(u\right)}^{s}\text{d}u,\\ E_{i:n}\left(x^{s}\right)=\frac{1}{B\left(i,n-i+1\right)}\overset{\infty }{\underset{r,t=0}{\sum }}\overset{n-i}{\underset{h=0}{\sum }}{\overset{⌣}{z}}_{r}c_{t,h+i-1}{\left(-1\right)}^{h}\left(\begin{array}{c} n-i\\ h \end{array}\right)\int_{0}^{1}u^{r+t}Q{\left(u\right)}^{s}\text{d}u. \end{array}$$

Thus, the mgf and other properties for EGIKw-G order statistics can also be obtained likewise.

## 5. Estimation

We employ MLE for estimating unknown parameters of EGIKw-G distribution. Let *ϑ* be *p*-dimensional baseline parametric vector. Consider *rv* 's *X*_1_, *X*_2_ …, *X*_*n*_, with each *X*_*i*_ coming from a EGIKw-G (*α*, *β*, *γ*, *λ*, *ϑ*)′ model. The log-likelihood *l*=*l*(Θ) is obtained from (5) as follows:

(74) $$\begin{array}{c} l\left(\Theta \right)=n\;\log\left(\alpha \beta \gamma \lambda \right)+\overset{n}{\underset{i=1}{\sum }}\log w\left(x_{i},\vartheta \right)\\ +\left(\gamma \lambda -1\right)\overset{n}{\underset{i=1}{\sum }}\log W\left(x_{i},\vartheta \right)-\left(\gamma +1\right)\overset{n}{\underset{i=1}{\sum }}\log\left[1-W^{\lambda }\left(x_{i},\vartheta \right)\right]-\left(\alpha +1\right)\overset{n}{\underset{i=1}{\sum }}\log\left[1+{\left(W^{-\lambda }\left(x_{i},\vartheta \right)-1\right)}^{-\gamma }\right]\\ +\left(\beta -1\right)\overset{n}{\underset{i=1}{\sum }}\log\left[1-{\left[1+{\left(W^{-\lambda }\left(x_{i},\vartheta \right)-1\right)}^{-\gamma }\right]}^{-\alpha }\right]. \end{array}$$

The components of *U*=(*U*_*α*_, *U*_*β*_, *U*_*γ*_, *U*_*λ*_, *U*_*ϑ*_)′, the score vector, are

(75) $$\begin{array}{c} U_{\alpha }=\frac{n}{\alpha }-\overset{n}{\underset{i=1}{\sum }}\log\left[1+{\left(W^{-\lambda }\left(x_{i},\vartheta \right)-1\right)}^{-\gamma }\right]\\ +\left(\beta -1\right)\overset{n}{\underset{i=1}{\sum }}\frac{\log\left[1+{\left(W^{-\lambda }\left(x_{i},\vartheta \right)-1\right)}^{-\gamma }\right]}{1-{\left[1+{\left(W^{-\lambda }\left(x_{i},\vartheta \right)-1\right)}^{-\gamma }\right]}^{\alpha }},\\ U_{\beta }=\frac{n}{\beta }+\overset{n}{\underset{i=1}{\sum }}\log\left[1-{\left[1+{\left(W^{-\lambda }\left(x_{i},\vartheta \right)-1\right)}^{-\gamma }\right]}^{-\alpha }\right],\\ U_{\gamma }=\frac{n}{\gamma }+\lambda \overset{n}{\underset{i=1}{\sum }}\log W\left(x_{i},\vartheta \right)\\ -\overset{n}{\underset{i=1}{\sum }}\log\left[1-W^{\lambda }\left(x_{i},\vartheta \right)\right]+\left(\alpha +1\right)\overset{n}{\underset{i=1}{\sum }}\frac{\log\left(W^{-\lambda }\left(x_{i},\vartheta \right)-1\right)}{1+{\left(W^{-\lambda }\left(x_{i},\vartheta \right)-1\right)}^{\gamma }}\\ +\left(\beta -1\right)\alpha \overset{n}{\underset{i=1}{\sum }}\frac{\log\left(W^{-\lambda }\left(x_{i},\vartheta \right)-1\right)}{\left[1-{\left(1+{\left(W^{-\lambda }\left(x_{i},\vartheta \right)-1\right)}^{-\gamma }\right)}^{\alpha }\right]\left[1+{\left(W^{-\lambda }\left(x_{i},\vartheta \right)-1\right)}^{\gamma }\right]},\\ U_{\lambda }=\frac{n}{\lambda }+\gamma \overset{n}{\underset{i=1}{\sum }}\log W\left(x_{i},\vartheta \right)-\left(\gamma +1\right)\overset{n}{\underset{i=1}{\sum }}\frac{\log\;W\left(x_{i},\vartheta \right)}{\left(W^{-\lambda }\left(x_{i},\vartheta \right)-1\right)}\\ +\left(\alpha +1\right)\gamma \overset{n}{\underset{i=1}{\sum }}\frac{\log\;W\left(x_{i},\vartheta \right)\left(W^{-\lambda }\left(x_{i},\vartheta \right)-1\right)}{W^{\lambda }\left(x_{i},\vartheta \right)\left[1+{\left(W^{-\lambda }\left(x_{i},\vartheta \right)-1\right)}^{\gamma }\right]}\\ +\left(\beta -1\right)\alpha \gamma \overset{n}{\underset{i=1}{\sum }}\frac{\log\;W\left(x_{i},\vartheta \right)\left[W^{-\lambda }\left(x_{i},\vartheta \right)-1\right]{\left[1+{\left(W^{-\lambda }\left(x_{i},\vartheta \right)-1\right)}^{\gamma }\right]}^{-1}}{W^{\lambda }\left(x_{i},\vartheta \right)\left[1-{\left[1+{\left(W^{-\lambda }\left(x_{i},\vartheta \right)-1\right)}^{-\gamma }\right]}^{\alpha }\right]},\\ U_{\vartheta }=\overset{n}{\underset{i=1}{\sum }}\frac{w{\;}^{\prime }\left(x_{i},\vartheta \right)}{w\left(x_{i},\vartheta \right)}\\ +\left(\gamma \lambda -1\right)\overset{n}{\underset{i=1}{\sum }}\frac{W{\;}^{\prime }\left(x_{i},\vartheta \right)}{W\left(x_{i},\vartheta \right)}+\lambda \left(\gamma +1\right)\overset{n}{\underset{i=1}{\sum }}\frac{W^{\prime }\left(x_{i},\vartheta \right)}{W\left(x_{i},\vartheta \right)\left(W^{-\lambda }\left(x_{i},\vartheta \right)-1\right)}\\ -\left(\alpha +1\right)\gamma \lambda \overset{n}{\underset{i=1}{\sum }}\frac{W{\;}^{\prime }\left(x_{i},\vartheta \right)}{W\left(x_{i},\vartheta \right)\left(1-W^{\lambda }\left(x_{i},\vartheta \right)\right)\left[1+{\left(W^{-\lambda }\left(x_{i},\vartheta \right)-1\right)}^{\gamma }\right]}\\ -\left(\beta -1\right)\alpha \gamma \lambda \overset{n}{\underset{i=1}{\sum }}\frac{W{\;}^{\prime }\left(x_{i},\vartheta \right){\left[1-{\left({\left(W^{-\lambda }\left(x_{i},\vartheta \right)-1\right)}^{-\gamma }+1\right)}^{\alpha }\right]}^{-1}}{W\left(x_{i},\vartheta \right)\left(1-W^{\lambda }\left(x_{i},\vartheta \right)\right)\left[1+{\left(W^{-\lambda }\left(x_{i},\vartheta \right)-1\right)}^{\gamma }\right]}. \end{array}$$

By solving *U*_*α*_=0, *U*_*β*_=0, *U*_*γ*_=0, *U*_*λ*_=0, and *U*_*ϑ*_=0, we obtain the MLEs $\left(\hat{\alpha },\hat{\beta },\hat{\gamma },\hat{\lambda },\hat{\vartheta }\right)$.

## 6. Monte Carlo Simulation

In this part, we examined the usefulness of MLEs for EGIKw-Burr XII (a special model from the family) parameters, through an extensive numerical investigation. Average bias (AB) and root mean square error (RMSE) are considered to evaluate the performance of estimators for varying *n*, *s*. The qf given by (7) with Burr XII as baseline model was considered for generating EGIKw-Burr XII rv. The simulation was repeated 2,000 times for varying samples. Four different parametric values, $\begin{array}{c} I:\left(\alpha =1.3,\beta =0.1,\gamma =1.5,\lambda =0.2,\psi =1.25,\xi =5.0\right),II:\left(\alpha =0.2,\beta =0.5,\gamma =0.15,\lambda =0.25,\psi =3.3,\xi =2.5\right),\\ III:\left(\alpha =2.0,\beta =0.6,\gamma =1.5,\lambda =1.75,\psi =0.45,\xi =4.0\right),IV:\left(\alpha =1.75,\beta =0.25,\gamma =1.5,\lambda =0.05,\psi =0.7,\xi =2.25\right) \end{array}$, were considered. The MLEs, AB, and RMSE values for different *n*, *s* are presented in Tables 1 and 2. From the results, it is clear that as *n* increases, the RMSE for estimators on the average decreases. It is also observed that for all four sets, the AB showed decreasing pattern as *n* increases. Thus, MLE method performs quite well in parameter estimation of proposed *G* class.

## 7. Application

In this part of work, we use EGIKw-Burr XII distribution for cancer patients' data to illustrate the merit of GIKw-Burr XII model compared to the generalized inverted Kumaraswamy (GIKw-Burr XII) by Jamal et al. (2019), the exponentiated Kumaraswamy Burr XII (EKwBXII) distributions by Paranaiba et al. (2013), the inverse Weibull Burr XII (IW-Burr XII) Model by Amal et al. (2018), the exponentiated Kumaraswamy Burr XII (EK-Burr XII) distribution by Silva et al. (2019), the exponentiated Weibull distribution (EWD) by Nassar and Fissa (2003), the generalized inverse Weibull distribution (GIWD) by De Gusmao et al. (2011), the modified extension of exponential (MEXED) distribution by El-Damcese and Ramadan (2015), and the well-known Burr XII distribution.

For each considered model, we obtain the estimates using MLE method and adopt the minimum value of -log(likelihood) at MLE denoted by (−*l*), Akaike Information Criterion (AIC), Bayesian Information Criterion (BIC), Consistent Akaike Information Criterion (CAIC), Hannan–Quinn Information Criterion (HQIC), Anderson-Darling (*A*^*∗*^) statistics, Cramér–von Mises (*W*^*∗*^) statistics, and Kolmogorov–Smirnov (K-S) tests. Data is about remission times of 128 bladder cancer patients in months from Lee and Wang (2003) and is provided as follows:

6.94, 8.66, 0.08, 2.09, 3.48, 4.87, 13.11, 23.63, 0.20, 9.02, 13.29, 0.40, 2.23, 3.52, 4.98, 6.97, 2.26, 3.57, 5.06, 7.09, 7.26, 9.47, 14.24, 25.82, 0.51, 2.54, 3.70, 5.17, 7.28, 9.74, 14.76, 14.77, 32.15, 2.64, 3.88, 5.32, 7.39, 10.34, 14.83, 34.26, 0.90, 2.69, 26.31, 9.22, 13.80, 25.74, 0.50, 2.46, 3.64, 5.09, 10.66, 15.96, 36.66, 1.05, 2.69, 4.23, 5.41, 7.62, 10.75, 16.62, 0.81, 2.62, 3.82, 5.32, 7.32, 10.06, 79.05, 4.18, 5.34, 7.59, 43.01, 1.19, 2.75, 4.26, 5.41, 7.63, 17.12, 1.26, 2.83, 4.33, 5.49, 7.66, 4.34, 5.71, 7.93, 11.79, 11.25, 17.14, 1.35, 2.87, 5.62, 7.87, 11.64, 17.36, 1.76, 3.25, 4.50, 6.25, 8.37, 12.02, 2.02, 3.31, 4.51, 6.54, 8.53, 12.03, 20.28, 2.02, 1.40, 3.02, 18.10, 1.46, 4.40, 5.85, 8.26, 3.36, 6.93, 8.65, 11.98, 19.13, 3.36, 6.76, 12.07, 21.73, 2.07, 12.63, 22.69.

**Figure 5:**
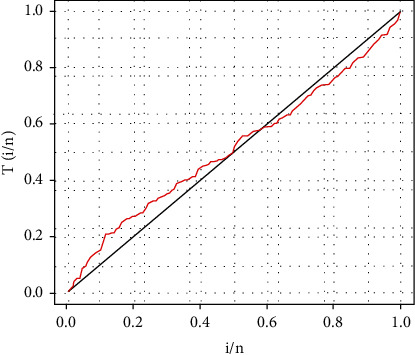
TTT-transform plot for cancer patients' data set.

The key statistics of data are offered in Table 3. Furthermore, the TTT- transform curve is depicted by Figure 5, which suggests an upside down bathtub or unimodal failure rate and, therefore, indicates that the EGIKw-Burr XII distribution is suitable for fitting this data set.

**Figure 6:**
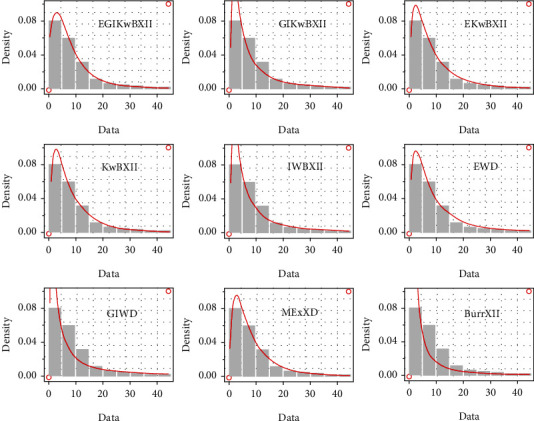
The pdf of considered models.

**Figure 7:**
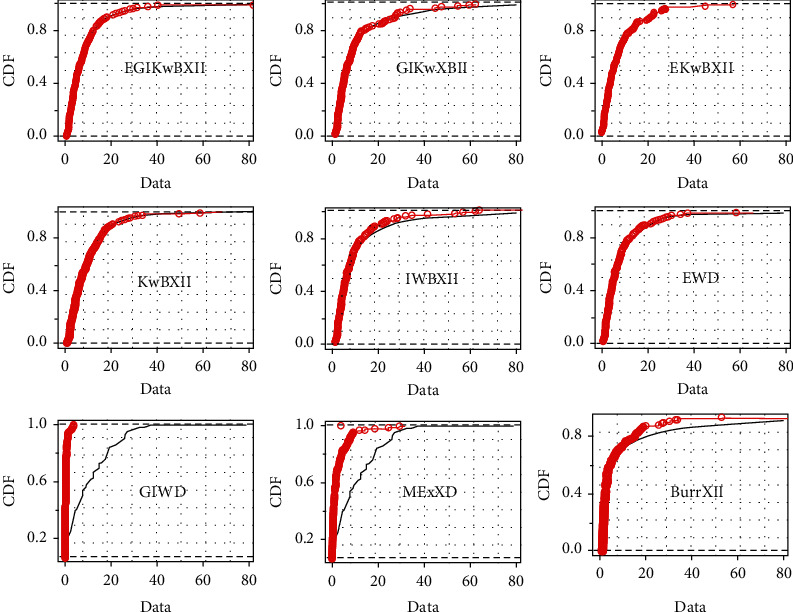
The cdf of considered models.

**Figure 8:**
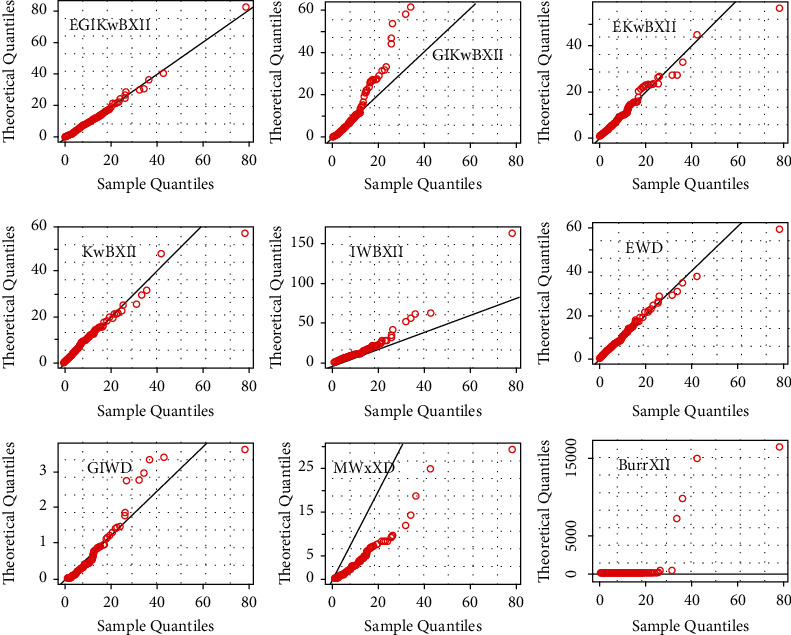
QQ-plots of considered distributions.

**Figure 9:**
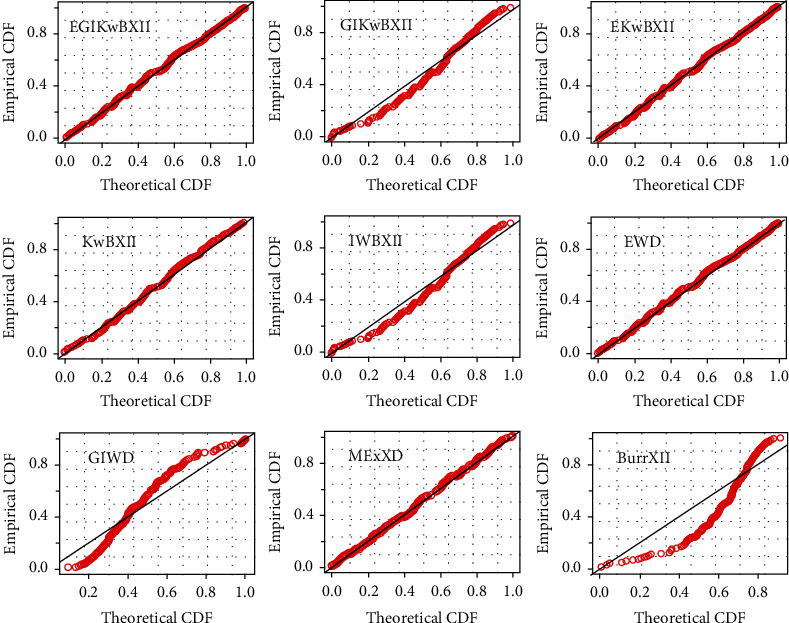
PP-plots of considered distributions.

Table 4 gives MLEs and standard error (SE) (within parentheses) results. The computed goodness-of-fit (gof) results are provided in Table 5. Histograms with estimated pdf plot, cdf plot, QQ-plot, and PP-plot of the EGIKw-Burr XII and other distributions are provided in Figures 6–9, respectively. It is clear from these results that EGIKw-Burr XII model with six parameters offers a better fit than other distributions.

## 8. Conclusions

In this work, a four-parameter generated class of models, EGIKw-G class, is proposed. Submodels of the proposed class, namely, the EGIKw-Normal, EGIKw-Fréchet, EGIKw-Uniform, and the EGIKw-Burr XII distributions, are discussed. Various properties including sf, hrf, rhrf, qf and median, *s*^th^ raw moment, mgf, md, Rényi entropy, reliability parameter, Lorenz and Bonferroni curves, residual lifetime, and distribution of order statistics are presented. Particular attention is given to EGIKw-Burr XII distribution. A MCS is presented to investigate the performance of AB and RMSE of MLEs. A real application is provided to check the usefulness of EGIKw-G class and its performance compared to other well-known distributions. The gof measures used all revealed that the novel model performed better than its counterparts [29,30].

## Figures and Tables

**Table 1:** Mean estimates, AB, and RMSEs of EGIKw-Burr XII distribution for some parameter values.

*n*	Par	I			II		
		MLE	RMSE	AB	MLE	RMSE	AB

25	*α*	2.031369	0.782508	−0.73137	2.050077	1.869672	−1.85008
	*β*	0.506018	0.426535	−0.40602	0.523979	0.111397	−0.02398
	*γ*	1.539714	0.101712	−0.03971	1.536192	1.391208	−1.38619
	*λ*	1.679459	1.620032	−1.47946	1.721563	1.589122	−1.47156
	*ψ*	0.513995	0.749618	0.736005	0.495447	2.80828	2.804553
	Ξ	5.536725	2.076617	−0.53672	5.608749	3.802548	−3.10875

50	*α*	2.003241	0.732436	−0.70324	2.027523	1.835541	−1.82752
	Β	0.4953	0.406724	−0.3953	0.511869	0.073648	−0.01187
	*γ*	1.516571	0.060813	−0.01657	1.519474	1.370756	−1.36947
	*λ*	1.553486	1.408353	−1.35349	1.598642	1.388082	−1.34864
	*ψ*	0.514657	0.745424	0.735343	0.49416	2.805931	2.80584
	*ξ*	5.140693	1.181488	−0.14069	5.230354	2.985746	−2.73035

100	*α*	1.999447	0.71545	−0.69945	2.011497	1.815691	−1.8115
	*β*	0.497415	0.403892	−0.39741	0.505193	0.051041	−0.00519
	*γ*	1.508029	0.040566	−0.00803	1.508947	1.359594	−1.35895
	*λ*	1.519543	1.347079	−1.31954	1.544223	1.312065	−1.29422
	*ψ*	0.51066	0.746059	0.73934	0.497226	2.80282	2.802774
	*ξ*	5.059594	0.784378	−0.05959	5.06391	2.690264	−2.56391

200	*α*	2.001363	0.710236	−0.70136	2.009553	1.811641	−1.80955
	*β*	0.497136	0.400706	−0.39714	0.50296	0.03563	−0.00296
	*γ*	1.504634	0.029312	−0.00463	1.505497	1.35581	−1.3555
	*λ*	1.513101	1.327808	−1.3131	1.52703	1.285339	−1.27703
	*ψ*	0.50596	0.747874	0.74404	0.498405	2.801618	2.801595
	*ξ*	5.000164	0.562188	−0.00016	5.025616	2.588476	−2.52562

**Table 2:** Mean estimates, AB, and RMSEs of EGIKw-Burr XII distribution for some parameter values.

*n*	Par	III			IV		
		MLE	RMSE	AB	MLE	RMSE	AB

25	*α*	2.052153	0.253051	−0.05215	2.055445	0.391286	−0.30544
	*β*	0.519755	0.132899	0.080246	0.516816	0.286654	−0.26682
	*γ*	1.538797	0.099868	−0.0388	1.539888	0.101188	−0.03989
	*λ*	1.712694	0.569639	0.037306	1.721858	1.76664	−1.67186
	*ψ*	0.488655	0.051269	−0.03865	0.488123	0.214635	0.211877
	*ξ*	5.542568	2.588892	−1.54257	5.512202	3.89718	−3.2622

50	*α*	2.014992	0.176476	−0.01499	2.03334	0.332539	−0.28334
	*β*	0.506935	0.117908	0.093065	0.511119	0.27147	−0.26112
	*γ*	1.515558	0.061392	−0.01556	1.520918	0.065155	−0.02092
	*λ*	1.577739	0.372816	0.172261	1.612466	1.599495	−1.56247
	*ψ*	0.495612	0.051045	−0.04561	0.493362	0.20797	0.206638
	*ξ*	5.133847	1.675164	−1.13385	5.250112	3.252078	−3.00011

100	*α*	2.017654	0.121994	−0.01765	2.009871	0.287532	−0.25987
	*β*	0.503799	0.108756	0.096201	0.5049	0.259768	−0.2549
	*γ*	1.510631	0.042348	−0.01063	1.50819	0.042492	−0.00819
	*λ*	1.555648	0.289758	0.194352	1.542143	1.507662	−1.49214
	*ψ*	0.496423	0.049195	−0.04642	0.497424	0.203226	0.202576
	*ξ*	5.073991	1.347996	−1.07399	5.052891	2.916263	−2.80289

200	*α*	2.00675	0.085296	−0.00675	2.006998	0.27192	−0.257
	*β*	0.5025	0.103631	0.097501	0.501314	0.25384	−0.25131
	*γ*	1.504946	0.028846	−0.00495	1.504844	0.029993	−0.00484
	*λ*	1.524199	0.267619	0.225801	1.52528	1.483044	−1.47528
	*ψ*	0.498184	0.049478	−0.04818	0.498387	0.201937	0.201613
	*ξ*	5.000275	1.141549	−1.00028	4.992079	2.797957	−2.74208

**Table 3:** Descriptive statistics.

*n*	Min.	Max.	Mean	Var.	Sd.	CV	Skew.	Kurt.

127	0.080	79.050	9.076	100.496	10.025	1.105	12.319	21.451

**Table 4:** MLE and SE.

Model	Estimates					

EGIKwBXII	1.43162 (1.4027)	0.68899 (0.50048)	1.54554 (3.69725)	11.05201 (31.96517)	1.16295 (2.92883)	0.14219 (0.55648)
GIKwBII	0.16048 (0.01133)	11.76763 (2.09854)	1.05116 (0.14219)	13.84875 (0.01808)	0.58321 (0.00246)	-
EKwBXII	0.36633 (0.42895)	0.97155 (1.67659)	10.15582 (16.18606)	44.13510 (61.61306)	0.81470 (0.56507)	-
KwBXII	0.31380 (0.35338)	0.96498 (1.84057)	9.81034 (17.57105)	68.76241 (98.86293)	-	-
IWBXII	0.97875 (0.24963)	3.02715 (0.54086)	91.44719 (53.15231)	0.40185 (0.08382)	-	-
EWD	0.68382 (0.13962)	2.62405 (1.15772)	0.27887 (0.14828)	-	-	-
GIWD	1.22766 (90.56746)	2.07078 (115.34209)	0.75502 (0.04268)	-	-	-
MExXD	0.33227 (0.04008)	0.08451 (0.03658)	0.09726 (0.06376)	-	-	-
BurrXII	2.33681 (0.35412)	0.23558 (0.04028)	-	-	-	-

**Table 5:** gof results.

Model	−*l*	AIC	CAIC	BIC	HQIC	*W* ^*∗*^	*A* ^*∗*^	K-S

EGIKwBXII	402.45120	811.26850	811.46360	819.80100	814.73520	0.01731	0.11223	0.03420 (0.9984)
GIKwBII	418.10950	846.21900	846.71480	860.43990	851.99670	0.35595	2.28401	0.09996 (0.1580)
EKwBXII	403.76400	817.52800	818.02380	831.74890	823.30580	0.04218	0.29185	0.04705 (0.9413)
KwBXII	403.69710	815.39430	815.72210	826.77100	820.01650	0.04093	0.28388	0.04576 (0.9531)
IWBXII	415.47480	838.94960	839.27740	850.32630	843.57180	0.27453	1.78637	0.10128 (0.1477)
EWD	403.52050	813.04110	813.23620	821.57360	816.50780	0.03961	0.26609	0.04446 (0.9633)
GIWD	437.75000	881.49990	881.69500	890.03250	884.96660	0.77033	4.69969	0.14576 (0.0090)
MExXD	402.63420	816.90240	817.60240	833.96750	823.83570	0.02090	0.13983	0.03537 (0.9981)
BurrXII	446.98390	897.96780	898.06460	903.65620	900.27900	0.77030	4.67668	0.25203 (0.0001)

## Data Availability

The data are included in the paper.
